# HD-FFQ to Detect Nutrient Deficiencies and Toxicities for a Multiethnic Asian Dialysis Population

**DOI:** 10.3390/nu12061585

**Published:** 2020-05-28

**Authors:** Mohammad Syafiq Md Ali, Zu-Wei Yeak, Ban-Hock Khor, Sharmela Sahathevan, Ayesha Sualeheen, Jun-Hao Lim, Nurul Iman Hafizah Adanan, Abdul Halim Abdul Gafor, Nor Fadhlina Zakaria, Pramod Khosla, Tilakavati Karupaiah, Zulfitri Azuan Mat Daud

**Affiliations:** 1 Department of Nutrition and Dietetics, Faculty of Medicine and Health Sciences, Universiti Putra Malaysia, Serdang 43400, Malaysia; mohammadsyafiqali14@gmail.com (M.S.M.A.); sinhao0624@yahoo.com (J.-H.L.); iman.hafizah@yahoo.com (N.I.H.A.); 2 School of Healthcare Science, Faculty of Health Sciences, Universiti Kebangsaan Malaysia, Jalan Raja Muda Abdul Aziz, Kuala Lumpur 50300, Malaysia; yeak_wei@hotmail.com (Z.-W.Y.); sham_0901@yahoo.com (S.S.); aishaltaf@ymail.com (A.S.); 3 Department of Medicine, Hospital Canselor Tuanku Muhriz, Universiti Kebangsaan Malaysia Medical Centre, Kuala Lumpur 56000, Malaysia; khorbanhock@gmail.com (B.-H.K.); halim@ppukm.ukm.edu.my (A.H.A.G.); 4 Department of Medicine, Faculty of Medicine and Health Sciences, Universiti Putra Malaysia, Serdang 43400, Malaysia; n_fadhlina@upm.edu.my; 5 Department of Nutrition and Food Science, Wayne State University, Detroit, MI 48202-0340, USA; aa0987@wayne.edu; 6 School of Biosciences, Taylors’ University, Subang Jaya 47500, Malaysia; tilly_karu@yahoo.co.uk; 7 Research Center of Excellence (RCoE) Nutrition and Non-communicable Diseases, Faculty of Medicine and Health Sciences, Universiti Putra Malaysia, Serdang 43400, Malaysia

**Keywords:** food frequency questionnaire, hemodialysis, dietary assessment, validity, multi-ethnic food intake

## Abstract

A rapid and reliable tool appropriate to quantifying macronutrient and micronutrient intakes in diets consumed by Malaysian hemodialysis (HD) patients is lacking. We aimed to develop and validate a novel HD-food frequency questionnaire (HD-FFQ) to assess habitual nutritional intakes of HD patients with diverse ethnic backgrounds. This study was conducted in three phases. In Phase I, a HD-FFQ comprising 118 food items was developed using 3-day diet recalls (3DDR) from 388 HD patients. Phase II was the face and content validation using the Scale-Content Validity Index (S-CVI). After successfully developing the FFQ, Phase III tested relative validation against a reference method, the 3DDR. Results from Phase III showed that the mean difference for absolute intakes of nutrients assessed by HD-FFQ and 3DDR were significant (*p* < 0.05). However, there was a significant correlation between the HD-FFQ and reference method ranging from 0.35–0.47 (*p* < 0.05). Cross-quartile classification showed that <10% of patients were grossly misclassified. In conclusion, the HD-FFQ has an acceptable relative validity in assessing and ranking the dietary intake of the HD patients in Malaysia.

## 1. Introduction

Dietary intake evaluation of a patient is a critical step of nutrition assessment in the nutrition care process pathway, but there is a major gap between this recommended task and its actual performance in outpatient dialysis settings in many parts of the world. The status of the global nephrology workforce reported by the Global Kidney Health Atlas [1] indicates a gross shortage of dietitian manpower by 78%, particularly afflicting Africa (91%) and Asia (80–92%). Although dietary assessment relies on dietary recall and food records from patients as shown in local studies on the Malaysian HD population [2,3,4], carrying out these methods requires a dietitian in order to ensure data quality [5]. In the context of the Malaysian outpatient HD setting, dietary assessment is rarely performed because of limited availability of dietitians [6]. Even in the United States, despite the presence of dietitians in the dialysis unit, in practice dietitians reported lack of time and tools to collect and analyze dietary information of HD patients [7]. 

Lack of dietary assessment hinders the identification and stratification of patients at risk and those requiring dietary intervention. HD patients on regular dialysis may be vulnerable to overhydration [8], hyperphosphatemia [9], and hyperkalemia [10] as well as muscle wasting resultant of protein energy wasting [11,12] whenever dietary energy intakes (DEI) and dietary protein intakes (DPI) are inadequate. The purpose of dietary assessment for the dialysis patient, therefore, is to detect malnutrition, nutrient deficiencies (i.e., energy, protein, water soluble vitamins) and toxicities (i.e., potassium and phosphate), and fluid excesses. Adequacy and compliance to nutrient goals are, therefore, critical outcomes that can be measured by performing regular dietary assessment. 

The food frequency questionnaire (FFQ) is an alternative option for the clinical setting as the patient and caregiver burdens are lower, with advantages from its characteristics such as its non-invasive nature, that it is time-saving, inexpensive, and potentially self-administered, and has the ability to capture typical dietary intake as shown in large-scale epidemiological studies [13,14,15]. As applied to the HD population, an FFQ has been developed and validated for American patients [16,17] but it is noteworthy that beyond macronutrient assessment, this tool does not detect patient risk for nutrient toxicities prevailing in HD patients. To date, no FFQ has been developed specific to the Malaysian HD population. Malaysia is an ethnically diverse country, with Malays, Chinese, and Indians consuming ethnic specific foods which are reflected in diverging food choices within food groups.

The development of a self-administered dialysis-specific FFQ, therefore, will be beneficial in the clinical setting to detect both nutrient deficiencies and nutrient toxicities. The tool is for both non-dietitian healthcare professionals as well as dietitians or used by the patients themselves to assess adequacy of dietary intake. Thus, the development and validation of an FFQ specific for the HD population appears to be critically needed taking into account the needs of ethnically diverse Malaysian HD patients. 

## 2. Materials and Methods 

This study comprised three phases; Phase I was the development of the HD-FFQ; whilst Phase II and III focused on its validation. 

### 2.1. PHASE I: Development of HD-FFQ

Dietary information from HD patients participating in two multi-center trials, namely Palm Tocotrienols in Chronic Hemodialysis and Hemodialysis Nutrition Study (HDiNS) was utilized in the development of a primary dietary database. The inclusion criteria for recruitment for the two trials were similar as per: Patients > 18 years old, been dialyzed for at least 3 months, and willing to provide information on their dietary intake. The dietary information was collected using the 3-day dietary recall (3DDR) method with 24-hour diet recalls collected for a dialysis day, a non-dialysis day, and a weekend. A total of 388 patients composed of three main ethnic groups (Malay, Chinese, and Indian) in Malaysia provided written informed consent for this study. Sociodemographic profile of Phase I subjects is provided as the supplementary material (Table S1). Ethical approval for this study was received from the Medical Research Ethic Committee, Ministry of Health, Malaysia (NMRR-14-1859-23386; NMRR-15-865-25260, respectively). 

Collection of 3DDRs from a total of 388 subjects yielded 1164 recalls, which were utilized to develop the food item list. The food items from the 3DDR were extracted into a Microsoft Office Excel 2013 sheet (Microsoft Corp., Redmond, WA). These food items were classified into food groups [18], namely (1) cooked rice; (2) noodles; (3) cereals and products; (4) meat, poultry, and products; (5) fish, shellfish, and products; (6) legumes; (7) vegetables; (8) fruits; (9) fast food; (10) milk and dairy products; (11) traditional *kuih*; (12) bakery and sweets; (13) snacks and finger foods; (14) jam and spread; (15) beverages; (16) sauces, condiments, and soup; and (17) health/oral nutrition supplements. The food listing comprising 14,049 food items was then merged according to similarity of foods within the same categories, and the frequency of each food item subsequently calculated, as described by Block et al. (1986). Various types of cooking methods for one specific food were counted as one food item. For example, stir-fried chicken, curry chicken, steamed chicken, and other chicken recipes were classified under the food item “chicken”. This process narrowed the total food items list to 329 food items. The merging process for each food item further grouped together similar foods, which basically shared a similar cooking method. For example, “chicken cooked with soy sauce”, “chicken cooked with ginger”, and “chicken cooked with onion” were grouped together as a subcategory under the food item “chicken”.

The inclusion of food items in the HD-FFQ were based on the criteria of been significant contributors to dietary energy and nutrients of interest (carbohydrate, protein, fat, sodium, potassium, phosphate, calcium, and iron) in this population’s food intake. Overall, the food items in the top 90% of contribution to the intake were shortlisted for the HD-FFQ [18]. The remaining 10% of the food items were dropped out from the list. The contribution of intake for each food item was calculated by using the formula below:

$$\%\;frequency\;intake\;contributed\;item,\;i=\frac{total\;intake\;of\;specific\;item,\;i}{total\;intake\;of\;all\;food}\times 100$$

This process yielded a total of 118 food items.

The database of food items was completed for total energy, carbohydrate, protein, fat, sodium, potassium, phosphate, calcium, and iron. The nutrient information was sourced from the Malaysian Food Composition Table, Singapore Food Composition Table, USDA National Nutrient Database and food labels [19,20,21]. Non-Malaysian databases were referred whenever the Malaysian food database did not provide information for specific foods. When no data was available from any database for specific food items, a standard recipe was constructed using Nutritionist Pro™ (Axxya Systems LLC, Stafford, TX, USA) software to complete the nutrient information. In addition, food labels of commercial food items were referred for nutrient information if such information was lacking in the food composition database. For foods that had no nutrient database or the recipe could not be developed, or without nutrition facts label, the food was substituted by similar types of food items.

Aside from building the nutrient database for the food items, information on the frequency of consumption and portion size was built into the HD-FFQ. The frequency of consumption of the food items was categorized as daily, weekly, monthly and rarely. The frequency option “rare” referred to food which only consumed in the past three months but less than once per month. Household measurements used as a reference were a small bowl, medium bowl, matchbox, glass, tablespoon and teaspoon.

The prototype of the HD-FFQ consisted of 118 food items categorized under 17 food groupings. (Supplementary file Table S2). The HD-FFQ included a patient instruction sheet. The format of this HD-FFQ was in a semi-quantitative form. 

### 2.2. PHASE II: Face and Content Validity of HD-FFQ

The face and content validity of HD-FFQ was tested with 10 HD patients as “laypersons” and 10 “experts” comprising dietitians and nephrologists. The number was based on recommendation from previous studies (between seven to ten people) to have sufficient control over chance agreement [22] whilst for content validity, the suggested number of subjects are between five to ten experts [23,24]. The laypersons recruited were literate, aged >18 years old, non-healthcare personnel and composed of three main ethnic groups. The characteristics for the experts included healthcare professionals: Eight dietitian and two nephrologists. The experts were experienced in managing HD patients, also comprised the three main ethnic groups. The duration to complete the HD-FFQ was timed and patients were required to provide their feedback on aspects including (i) identity/familiarity of the food items; (ii) portion size; (iii) relevance to dietary practice; (iv) clarity of questionnaire (layout, color, font size); and (v) flow of questionnaire using a five-point Likert scale (1 = very poor, 2 = poor, 3 = fair, 4 = good, and 5 = very good). The Scale-Content Validity Index (S-CVI) value was calculated based on the given score. The comments from the experts and laymen were compiled and documented. 

Amendments were made to the HD-FFQ based on suggestions from the experts and laypersons. The finalized HD-FFQ as a result of this process included 123 food items.

### 2.3. PHASE III: Relative Validity of HD-FFQ

Relative validation was carried out through a cross-sectional study with recruitment of HD patients based on Malaysia’s ethnic distribution (Malays, Chinese, and Indians). Patients involved in Phase I and Phase II study was excluded in this Phase. 

#### 2.3.1. Patients’ Recruitment

Patient screening was carried out in various dialysis units in the Klang Valley, Malaysia. Information such as sociodemographic characteristics, anthropometric measurements and biochemical profiles were collected through interview and medical records. This study received an ethical approval from the National Medical Research Ethics Committee (NMRR-18-2717-44019). Since this study was to evaluate the correlation of macro- and micronutrients assessed using HD-FFQ and 3DDR, the sample size formula which estimates total sample size based on correlation coefficient (r) value was used [25]. The correlation coefficient used was 0.4 based on the previous FFQ validation study conducted among Malay adolescent in Malaysia [26] with alpha value set at 0.05, power was 80% and non-response rate of 10%. The calculated sample size for this phase was 114 subjects.

The inclusion criteria were age > 18 years old, undergoing HD treatment thrice-weekly for >3 months whilst the exclusion criteria were patients with impaired mobility or physical function, not willing to follow the study protocol and were hospitalized during data collection.

#### 2.3.2. Biochemical Assessment

Biochemical profiles of the patients, that are routinely done quarterly, were obtained from their medical records. Typically, a mid-week pre-dialysis blood samples were collected from patients after 8-10 hours of fasting through dialysis access site into the BD Vacutainer® Serum Separator Tubes™. The blood collection procedure was handled by the dialysis nurse at the respective HD centers. The plasma samples were isolated by centrifugation, snap-frozen with liquid nitrogen and were sent to the external laboratory for processing. Routine blood tests assessed in this study included renal function test i.e., serum urea, creatinine, potassium, and phosphate. Briefly, serum urea was determined using urease-kinetic method, creatinine by Jaffee rate blanked compensated method, phosphorus by molybdate reagent method and potassium by enzymatic method using Cobas 6000 (Roche, Germany) as previously reported [27]. The assessment of these biomarkers was used as additional reference markers to validate the HD-FFQ.

#### 2.3.3. Dietary Assessment

Dietary intake assessment for each patient was collected using both the HD-FFQ and the reference method, which is 3DDR. For the 3DDR method, patients were approached to collect 24-h diet recalls reflecting a dialysis day, a non-dialysis day, and a weekend day. The recorded information included portion size, food preparation method, source of food, and brands of food if available. The serving size of food or beverages consumed was recorded based on household measurement. On the other hand, apart from explaining the written instructions on how to answer the HD-FFQ, the questionnaire was self-administered by the patients and counter-checked by the interviewers. For patients with poor literacy, the HD-FFQ was administered by the interviewers. 

#### 2.3.4. Analysis of Dietary Information from 3DDR and HD-FFQ

Dietary data from the 3DDRs were analyzed using the computer software Nutritionist Pro™ (Axxya Systems LLC, Stafford, TX, USA) by referencing the food item weight from the Malaysian Food Composition Table and Singapore Food Database [19,20]. Standard food recipes were constructed when the food item was not available in any either of the databases [28]. In addition, food labels of commercial food items were referred for nutrient information if such information was lacking in the food composition database. 

The HD-FFQ data were reported in terms of consumption frequency. The conversion factor for the frequency of intake of the food items are reported in a supplementary file (Table S3). If the reported frequency of consumption for a particular food item was given as a range number, an average value was taken by dividing the sum of minimum and maximum number in the range. The formula used to calculate daily nutrient intake from the FFQ [29] is as follows:

$$\begin{array}{l} Daily\;nutrient\;intake\\ \;=Conversion\;factor\times number\;of\;intake\times number\;of\;portion\;taken\\ \;\times weight\;of\;food\;per\;portion \end{array}$$

A tool was developed using Microsoft Office Excel to calculate the daily nutrient intake consumed by patients as reported in the HD-FFQ. The nutrient database used for the HD-FFQ data analysis was developed from the same database used for the 3DDR data analysis. 

#### 2.3.5. Misreporting

Mis-reporters were identified using the Energy Intake: Basal Metabolic Rate (EI: BMR) approach where an index value of <0.8 was classified as under-reporting, 0.8–2.4 was considered as acceptable-reporting and EI: BMR >2.4 categorized as over-reporting [30]. The cut-offs were identified based on physical activity level of 1.4 which indicates moderate-low physical activity [30]. Patients’ BMR was calculated by using the Harris–Benedict formula [31].

### 2.4. Statistical Analyses

Data analysis was performed using the Statistical Package for Social Sciences (SPSS) Software version 25. Prior to the analysis, the data was screened for outliers and missing values. Descriptive analysis was performed for all the parameters. The test for normality distribution was carried out for anthropometric, biochemical, and dietary data using Kolmogorov–Smirnov test. A *p*-value <0.05 was indicative of statistical significance. 

For Phase II data regarding validation of the HD-FFQ by experts and laypersons, Mann–Whitney U test was performed for mean differences of average scores as per food group. In addition, S-CVI was calculated to measure the overall validity of the content within the HD-FFQ [32]. Prior to the calculation of CVI, the score rating of the Likert scale was recoded as 1 (Score of ≥4) or 0 (Score of <4). The S-CVI is the proportion of items on the scale achieving a relevance score of ≥4 by all experts [33]. The acceptable cut-off score for CVI is ≥0.78 when >9 experts are involved [34]. 

For Phase III data, Pearson product–moment correlation tests were performed to determine the linearity between the HD-FFQ with the 3DDR and serum biomarkers. Correlation tests were adjusted for energy, age, gender, and body mass index (BMI). Paired *t*-test were used to compare the mean differences of nutrients between HD-FFQ and 3DDR. The graphical representation of the mean differences were shown in the Bland–Altman analysis (Supplementary file Figure S1). Finally, cross-quartile classification analysis [35] was used to observe whether the HD-FFQ method and the 3DDR method were able to discriminate patients to the correct quartile of nutrient intake level respectively (i.e., ranking the individual food intake). The acceptable cut-off for correlation test is when *p* < 0.05 [35] whilst the acceptable outcome for paired *t*-test is *p* > 0.05 [5]. For cross-quartile classification analysis, good outcome is considered when the percentage in opposite quartile (i.e., misclassification) is ≤10% [35].

To date, there is no “gold standard” statistical method in assessing the validity of the newly developed FFQs [35,36]. A review on the statistical test to evaluate the validity of dietary assessment method stated that there is no “fixed” number of statistical test that is compulsory to follow in a validation study to define absolute validity [36]. The common number of statistical test that was used in dietary assessment validation studies is between two to three statistical test [36]. In this study three types of statistical test were used and the term “acceptable relative validity” denotes that more than half of the completed statistical test meets the given cut-off criterion.

## 3. Results

### 3.1. PHASE II: Face and Content Validation of HD-FFQ

Face validation to test the HD-FFQ involved 10 experts (nephrologists = 2; dietitians = 8) and 10 multi-ethnic HD patients as lay persons. Mean recorded time to complete the HD-FFQ when self-administered by patients was 42.1 ± 5.2 min. Individual scores as per food groups were not significantly different between groups, except for “cooked rice” (*p* = 0.03). The highest median scores given by the experts observed in the “fruit” and “fish, shellfish and products” food group were 4.2 (4.0–4.8) and 4.2 (4.0–4.6), respectively. Meanwhile, the highest median score observed in laypersons group was seen in “cooked rice”, “noodles”, “meat, poultry and products”, “fruit”, and “fish, shellfish and products” food groups with similar median scores of 4.2. The individual scores achieved for each food group by expert and lay person groups are indicated in Table 1.

An additional test of S-CVI was performed within the expert group as per the scoring for the overall content of the HD-FFQ. The achieved score for S-CVI was 0.81, where the minimum score of >0.78 is considered acceptable showing good agreement between experts, in terms of the relevance of the food item within the HD-FFQ [32,33]. 

Comments from the validators, invited to improve the HD-FFQ or state its liked features, are provided as supplementary material (Table S4). An additional five food items were added in the prototype HD-FFQ, thus, the final number of food item listed in the HD-FFQ is 123 (Supplementary Material; Table S5). The final HD-FFQ form is also provided as Supplementary Material (Table S6).

### 3.2. PHASE III: Relative Validation of the HD-FFQ

Although 152 eligible patients were recruited for this phase, 31 patients were excluded in the final analysis as they were identified as mis-reporters as per EI: BMR category (Supplementary File; Table S7). 

#### 3.2.1. Study Population Characteristics

Patients (*n* = 121) had a mean age of 53 ± 12 years, with majority (29.5%) aged between 51 to 60 years old. The gender distribution was 55.4% male and 44.6% female. The ethnic distribution of patients was 63.7% Malay, 23.1% Chinese, and 13.2% Indian which approximately represented the three main ethnic groups residing in Klang Valley [37]. Majority of the patients (55.4%) had completed secondary school education, whilst 67.8% of the patients were not currently working. Using the criteria of the Report of Household Income and Basic Amenities Survey 2016 [38], 80.2% patients were classified as low income group (<RM 3000 per month). The mean dialysis vintage of the patients was 78 ± 60 months. All sociodemographic characteristics of Phase III patients are presented in Table 2. 

#### 3.2.2. Validity Tests between HD-FFQ and the 3DDR

a. Correlations between HD-FFQ and 3DDR

A correlation coefficient analysis by Pearson’s correlation was performed to determine the correlation of measured energy and nutrients between the HD-FFQ and 3DDR methods. Overall, significantly fair correlations were observed between the HD-FFQ and 3DDR. For unadjusted data, all nutrients are significantly correlated ranging from *r* = 0.35 for dietary sodium (mg/day) to the highest values of *r* = 0.51 for dietary calcium (mg/day) (Table 3). When the nutrient data were adjusted for energy intake, correlations of nutrients between the HD-FFQ and the 3DDR were found to be in the range of *r* = 0.19 for dietary sodium (mg/day) to *r* = 0.42 as for dietary calcium (mg/day) with iron been not significantly correlated. Additionally, energy and nutrients estimated via HD-FFQ remained significantly correlated with those of 3DDR when adjusted for additional co-variates such as age, gender and BM.

b. Correlations of HD-FFQ and 3DDR with serum biomarkers

The correlations of HD-FFQ and 3DDR with serum renal profile are presented in Table 4. HD-FFQ showed superior correlation with respective serum biomarkers of interest when compared to 3DDR. For example, dietary protein intake estimated using HD-FFQ had significant correlation (*p* < 0.05) with serum urea (*r* = 0.25) and creatinine (*r* = 0.22) but such correlations were not observed with 3DDR. Similarly, potassium and phosphate intakes estimated via HD-FFQ were significantly correlated with serum biomarkers of potassium and phosphate whereas these observations were not observed with 3DDR intake data. 

c. Mean difference between nutrients calculated as per HD-FFQ and 3DDR

Mean difference comparisons of dietary nutrients derived by both HD-FFQ and 3DDR methods are presented in Table 5. Percentage of mean differences ranged from 13.4% for carbohydrate (g/day) to 39.6% for potassium (mg/day). Paired sample *t*-test analysis between HD-FFQ and 3DDR dietary intake comparisons were statistically significant for all nutrients (*p* < 0.01).

d. Cross-quartile classification between HD-FFQ and 3DDR

The cross-quartile classification of dietary nutrients derived by HD-FFQ and 3DDR methods was checked as a measure of agreement between the two methods (Table 6). For nutrients classified within the same quartile, the lowest was for protein (29.8%) whilst the highest was for total energy and carbohydrate (both 40.5%). Nutrients classified in adjacent quartiles (±1 quartile apart) ranged from the lowest 33.9% for phosphate to the highest 42.1% for sodium. For nutrients that were grossly misclassified (±3 quartiles apart), the range was from the lowest 3.3% for carbohydrate and potassium to the highest 7.4% for sodium. Cross-classification of all the nutrients were found to be acceptable as gross misclassification was <10%.

## 4. Discussion

The finalised HD-FFQ with 123 food items is considered acceptable as it falls within the recommended numbers of food item for an ideal FFQ [39]. This HD-FFQ was designed to include composite meals defined by cooking techniques applicable to food group categories such as “meat, poultry and products”, “fish, shellfish and products”, “legumes”, “vegetables”, and “snacks and finger foods”. Composite meal-based FFQs have also been developed previously for some Asian countries [40,41] as Asian meals feature dishes with spices and seasonings (e.g., salt, turmeric, ginger, soy sauce, red pepper paste, and soybean paste) and sautéed in cooking oils (palm oil, coconut oil, soybean oil etc.), which together greatly contribute to nutrient intakes (e.g., energy, fat, sodium, and β-carotene intake). As such excluding them will underestimate these nutrients [42]. 

The average recorded time of 42.1 ± 5.2 min taken to complete the HD-FFQ is similar to previously validated FFQs [43,44]. Open comments from the experts agreed that the length of this HD-FFQ was acceptable. However, the HD-FFQ was considered lengthy by some patients, possibly reflecting low cognitive function and level of literacy [11]. Both expert and layperson groups well accepted the HD-FFQ, as indicated by non-significant median scores for majority of the food group except for the “cooked rice” food group. However, a statistically significant lower score was observed in “cooked rice” category attributed to respondent mis-classification between “plain white rice”, “fried rice”, “*nasi beriyani*” etc. However, the overall content validity of the newly developed HD-FFQ was good as evident by the calculated S-CVI score [45]. 

The correlation tests indicated the HD-FFQ effected acceptable outcomes for all of the study nutrients. As there is scarcity of comparator data in HD populations, the HD-FFQ validation was compared to non-CKD populations in Malaysia, and we found the correlation of unadjusted energy and macronutrients were in the range of 0.24 for fat to 0.72 for energy [46,47]. However, the strength of correlations for macro- and micronutrients decreased when adjusted for energy, and this trend is also reported by other validation studies [28,46,48]. The decrease in correlation value after adjustment for energy could be due to the systematic errors of overestimation or underestimation [49]. In addition, measurement error and within-person variability may partially contribute to the decreasing trend in the correlation values [50,51,52]. The measurement errors in the assessment of nutrient intakes are strongly correlated with errors in the measurement of total energy intake [53]. The purpose of energy-adjustment for both macro- and micronutrients is to alleviate the effects of measurement error in the data collection [54]. As previously reported, physiological characteristics were thought to affect the dietary intake of each individual [15,55]. Therefore, the nutrient intake was adjusted for age, gender, and BMI in order to detect the correlations between 3DDR and the HD-FFQ whilst controlling the influence of physiological and physical characteristics of the patients. Even though there is a slight decrease of correlation value when adjusted for age, gender and BMI, the *r*-value, however, is higher compared to the energy-adjusted value. From this, it can be concluded that the effect of these factors was minimal [56]. Additionally, the total energy intake has minimal variation due to well-regulated physiological mechanisms while individual macronutrients exhibit moderate variations towards contributing energy intake, therefore wider differences were observed between the unadjusted and energy-adjusted model [57].

In addition, we also examined the correlation of nutrients of interest estimated by HD-FFQ and 3DDR in relation to the blood biomarkers. Interestingly, the HD-FFQ had more numbers of nutrients that were significantly correlated with the serum renal profile when compared to the 3DDR. Although the serum biomarkers are affected by digestion, absorption, uptake, utilization, metabolism, excretion, and homeostatic mechanisms [39,58], the ability of HD-FFQ assessment to correlate with biomarkers will give health practitioners an insight into nutrient deficiencies and toxicities occurring because of dietary inadequacies, common to the HD population [59,60]. It can be concluded that this HD-FFQ ably captures micronutrient intakes due to the comprehensive food list included in the HD-FFQ, despite day-to-day variations in micronutrient consumption [15]. 

On the other hand, HD-FFQ significantly (*p* < 0.05) overestimates energy and nutrients intake as indicated by nutrients >10% for mean difference (MD). It is expected for a FFQ to overestimate energy and macronutrient intakes compared to the reference method [61,62,63]. Similar results reporting MD >10% for energy and macronutrients has been cited [29,64]. A local study among a multi-ethnic adolescent non-CKD population showed a significant difference (*p* < 0.05) between dietary carbohydrate and total fat, but total energy and other nutrients were not significantly different [47]. But another study among healthy Malay and Indian women observed that energy, macronutrients and vitamins did not significantly differ (*p* > 0.05) with an MD of <10% [65]. Conflicting results between studies reflect differences in reference methods used, variability of FFQ structure, sample size as well as differences in the populations being studied [66]. Apart from this, patient fatigue, patient burden, monotonous eating pattern, and misinterpreting food portion sizes may hinder accuracy of nutrient recall data [63,66,67].

Cross classification gives a clearer picture on how well the FFQ pairs with 3DDR, and also assesses the ability of the FFQ and 3DDR methods to classify the same individuals within the same categories of nutrient intake [15,36]. This study revealed a good agreement at individual level as gross misclassification (GM) values were <10%, as also found in other studies. GM values exceeding 10% are indicative of large within and between-person variation of intake in a study population [28].

The major strength of this study is the inclusion of a comprehensive database on commonly consumed foods representative of multi-ethnic communities in Malaysia, which is the way patients also consume diets. Besides that, the HD-FFQ was developed in bi-languages of “English” and “Bahasa Malaysia”, minimizing the implications of language barriers, particularly promoting self-administration in future patient use. Moreover, this HD-FFQ detailed preparation methods accompanying composite meals, which makes the instrument able to better quantify specific nutrients associated with the cooking method.

Some limitations are noted in this study. Firstly, the use of 3DDR as the main dietary reference method may not be the best reference method as it shares a similar bias (e.g., portion size estimation) with the test instrument. The weighed food record is a better reference method [68] as it is without recall bias. However, the use of weighed food record requires highly motivated patients and imposes a high burden to the patient, and is therefore not feasible to use in this population. As such, the National Kidney Foundation K/DOQI clinical practice guideline recommends, 3DDR as a practical tool to assess patients’ dietary intake [12]. To address this limitation, we compared serum concentrations of several biomarkers including potassium, phosphate, urea, and creatinine as biological reference to the nutrient intakes of interest, with the view that these biomarkers are highly affected by the disease pathophysiology.

In addition, due to the time constraint and because the main focus of this study was to develop and test the relative validity of the HD-FFQ with the reference method, no reliability test was administered to assess the reproducibility of the instrument. Several studies have also reported similar approaches in the development and validity of their FFQs with other reference methods prior to proceeding with reproducibility testing [69,70,71,72].

As such, it is recommended that for future studies that utilizing this HD-FFQ should include the reliability test and adaptation of the instrument with a ready calculator and progressing to an online or digitalized application to enable easy access for health practitioners and patients, and to promote patient self-efficacy.

## 5. Conclusions

In conclusion, the HD-FFQ had acceptable relative validity when compared with 3DDR based on the significant correlations with measured nutrients and good agreement with less misclassification. It is a suitable tool to assess and rank the habitual dietary intake of multi-ethnic Malaysian HD patients satisfactorily, and to correctly rank low and high intakes of energy, macro- and micronutrients which are relevant to the HD treatment and nutrient recommendation goals. We expect that this HD-FFQ is an appropriate tool to help clinicians identify and stratify patients at risk for nutrient deficiencies and toxicities to enable therapeutic interventions.

## Figures and Tables

**Table 1:** Individual food group scores as per experts and lay persons.

Food Groups	Median Score ^a^		*p*-Value ^b^
	Experts (*n* = 10)	Lay Person (*n* = 10)	
Cereals and Products	4.0 (4.0–4.4)	4.0 (3.4–4.3)	ns
Cooked Rice	4.0 (3.8– 4.3)	4.4 (4.0–4.7)	0.03 *
Noodles	4.0 (4.0–4.2)	4.4 (4.0–4.6)	ns
Traditional Kuih	4.1 (3.8–4.5)	4.0 (3.9–4.4)	ns
Bakery and Sweets	4.0 (4.0–4.3)	4.1 (3.8–4.4)	ns
Snacks and Finger Foods	4.0 (3.9–4.6)	3.9 (3.6–4.5)	ns
Meat, Poultry and Products	4.1 (4.0–4.5)	4.4 (3.9–4.7)	ns
Fruit	4.2 (4.0–4.8)	4.4 (4.2–4.7)	ns
Jam and Spread	4.1 (4.0–4.7)	4.2 (3.8–4.6)	ns
Beverages	4.1 (3.8–4.7)	4.2 (4.0–4.6)	ns
Sauces, Condiments and Soups	4.0 (4.0–4.3)	4.1 (4.0–4.3)	ns
Health Supplements	4.0 (3.8–5.0)	3.8 (3.5–4.1)	ns
Fast Food Chain	4.0 (4.0–4.7)	4.0 (3.8–4.5)	ns
Fish, Shellfish and Products	4.2 (4.0–4.6)	4.4 (3.8–5.0)	ns
Legumes	4.0 (4.0–4.3)	4.0 (3.5–4.4)	ns
Vegetables	4.1 (4.0–4.7)	4.0 (3.8–4.5)	ns
Milk and Dairy Products	4.0 (4.0–5.0)	3.9 (3.8–4.1)	ns

^a^ Median score presented as median (Interquartile range; Q1–Q3); ^b^ Mann–Whitney U test between groups; * significant if *p* < 0.05; ns, not significant.

**Table 2:** Sociodemographic characteristics of Phase III patients.

Characteristics (*n* = 121)	*n* (%)
Age (years), mean ± SD 20–30 years old 31–40 years old 41–50 years old 51–60 years old >60 years old	53 ± 12 4 (3%) 18 (13.6%) 24 (18.2%) 39 (29.5%) 36 (27.3%)
Dialysis vintage (months), mean ± SD	78 ± 60
Gender Male Female	67 (55.4) 54 (44.6)
Ethnicity Malay Chinese Indian	77 (63.7) 28 (23.1) 16 (13.2)
Educational level No formal education	1 (0.8)
Primary Secondary Tertiary (College/University)	20 (16.5) 67 (55.4) 33 (27.3)
Marital status Single Married Others	22 (18.2) 97 (80.2) 2 (1.7)
Working status Yes No	39 (32.3) 82 (67.8)
Income level ^a^ Low Income (Less than RM 3000) Medium Income (RM 3000 to RM 6275) High Income (More than RM 6275)	97 (80.2) 13 (10.7) 11 (9.1)
Alcohol drinker Yes No	4 (3.3) 117 (96.7)

Note: Data are presented as percentage (%) unless stated as mean ± SD. ^a^ Based on income level classification [38]. Abbreviations: ESRD: End-Stage Renal Disease, CVD: Cardiovascular Disease.

**Table 3:** Correlations between hemodialysis-food frequency questionnaire (HD-FFQ) and 3-day diet recall (3DDR) methods as per nutrients.

Nutrients	Unadjusted		Energy-Adjusted		Age, Gender and BMI-Adjusted	
	*r*	*p*-Value	*r*	*p*-Value	*r*	*p*-Value
Energy (Kcal/day)	0.46	<0.01	-	-	0.35	<0.01
Carbohydrate (g/day)	0.43	<0.01	0.31	<0.05	0.35	<0.01
Protein (g/day)	0.40	<0.01	0.32	<0.01	0.27	<0.05
Fat (g/day)	0.38	<0.01	0.33	<0.01	0.34	<0.01
Sodium (mg/day)	0.35	<0.01	0.19	<0.05	0.27	<0.05
Potassium (mg/day)	0.40	<0.01	0.20	<0.05	0.28	<0.05
Phosphate (mg/day)	0.37	<0.01	0.20	<0.05	0.29	<0.05
Calcium (mg/day)	0.51	<0.01	0.42	<0.01	0.45	<0.01
Iron (mg/day)	0.39	<0.01	0.01	ns	0.29	<0.05

Note: Correlation for unadjusted value for HD-FFQ and 3DDR was performed using Pearson’s correlation. Correlation test for adjusted values (Energy-adjusted and age, gender and body mass index (BMI)-adjusted) was performed using partial correlation coefficient). *p*-value < 0.05; ns, not significant.

**Table 4:** Correlations of HD-FFQ and 3DDR with serum renal profile.

Nutrients	Renal Profile															
	Serum Urea				Serum Creatinine				Serum Potassium				Serum Phosphate			
	HD-FFQ		3DDR		HD-FFQ		3DDR		HD-FFQ		3DDR		HD-FFQ		3DDR	
	*r*	*p* *^a^*	*r*	*p* *^a^*	*r*	*p* *^a^*	*r*	*p* *^a^*	*r*	*p* *^a^*	*r*	*p* *^a^*	*r*	*p* *^a^*	*r*	*p* *^a^*
Energy	0.09	ns	0.03	ns	0.14	ns	0.07	ns	0.19	<0.05	0.04	ns	0.18	<0.05	0.04	ns
Carbohydrate	−0.04	ns	−0.05	ns	0.08	ns	0.07	ns	0.11	ns	0.02	ns	0.09	ns	−0.02	ns
Protein	0.25	<0.01	0.15	ns	0.22	<0.05	0.08	ns	0.15	ns	0.09	ns	0.26	<0.01	0.11	ns
Fat	0.17	ns	0.08	ns	0.16	ns	0.02	ns	0.29	<0.01	0.03	ns	0.23	<0.05	0.07	ns
Sodium	0.23	<0.05	0.05	ns	0.16	ns	0.07	ns	0.23	<0.05	0.13	ns	0.28	<0.01	0.13	ns
Potassium	0.15	ns	0.13	ns	0.10	ns	0.11	ns	0.22	<0.05	0.002	ns	0.26	<0.01	0.14	ns
Phosphate	0.18	<0.05	0.06	ns	0.16	ns	0.04	ns	0.18	ns	0.06	ns	0.22	<0.05	0.02	ns
Calcium	0.16	ns	−0.05	ns	0.10	ns	0.02	ns	0.29	<0.01	0.20	<0.05	0.21	<0.05	0.03	ns
Iron	0.14	ns	0.08	ns	0.13	ns	0.06	ns	0.25	<0.01	0.11	ns	0.17	ns	0.11	ns

^a^ Pearson’s correlation, *p*-value < 0.05; Note: ns, not significant; HD-FFQ, Hemodialysis-Food Frequency Questionnaire; 3DDR, 3-Day Diet Recall

**Table 5:** Mean dietary nutrient intake and mean differences between HD-FFQ and 3DDR.

**Dietary Intake**	HD-FFQ	3DDR	Mean Difference	% Mean Difference ^b^	*p*-Value ^a^
	Mean (SD)	Mean (SD)			
Energy (kcal/day)	1826 (649)	1555 (401)	271	17.4	<0.01
Carbohydrate (g/day)	262 (91)	231 (64)	31	13.4	<0.01
Protein (g/day)	68 (29)	53 (19)	15	28.3	<0.01
Fat (g/day)	58 (27)	47 (20)	11	23.4	<0.01
Sodium (mg/day)	3015 (1375)	2395 (890)	620	25.9	<0.01
Potassium (mg/day)	1526 (613)	1093 (421)	433	39.6	<0.01
Phosphate (mg/day)	860 (361)	643 (241)	217	33.7	<0.01
Calcium (mg/day)	410 (181)	313 (171)	97	31.0	<0.01
Iron (mg/day)	14 (6)	11 (5)	3	27.3	<0.01

^a^ Paired sample *t*-test; between HD-FFQ and 3DDR, *p*-value > 0.05; ^b^ Percentage mean difference was individually computed using the formula (HD-FFQ–3DDR)/3DDR × 100.

**Table 6:** Cross-quartile classification of nutrients derived by HD-FFQ and 3DDR methods.

Nutrients	Same Quartile (%)	Adjacent Quartile (%)	Grossly Misclassified (%)
Energy (kcal/day)	40.5	36.4	6.6
Carbohydrate (g/day)	36.4	36.4	3.3
Protein (g/day)	29.8	39.7	5.0
Fat (g/day)	36.4	40.5	5.8
Sodium (mg/day)	33.9	42.1	7.4
Potassium (mg/day)	36.4	38.0	3.3
Phosphate (mg/day)	36.4	33.9	5.8
Calcium (mg/day)	40.5	41.3	5.0
Iron (mg/day)	36.4	39.7	5.0

Abbreviations: HD-FFQ, Hemodialysis-Food Frequency Questionnaire; 3DDR, 3-Day Diet Recall.

## Supplementary Materials

The following are available online at https://www.mdpi.com/2072-6643/12/6/1585/s1, Table S1: Patient Characteristics in Phase I, Table S2: Food item listing in the prototype HD-FFQ, Table S3: Conversion factor for the frequency of food item intake, Figure S1: Bland–Altman plot for dietary intakes derived by HD-FFQ and 3DDR methods, Table S4: Summary of comments from the expert and lay person, Table S5: Food item listing in the final HD-FFQ, Table S6: HD-FFQ form, Table S7: Identification of misreporters according to EI: BMR category.
