# Supplementary material for: HD-FFQ to Detect Nutrient Deficiencies and Toxicities for a Multiethnic Asian Dialysis Population
Source: Nutrients. 2020 May 28;12(6):1585. doi: 10.3390/nu12061585 (PMC7352606; doi:10.3390/nu12061585)
Supplement: Supplementary file 1 [file nutrients-12-01585-s001.pdf]

Table S1: Patient Characteristics in Phase I

**Patients characteristics**	**All (n=388)**
Age (year)	54 ± 15
Gender, n (%)	
Male	213 (54.9)
Female	175 (45.1)
Ethnic group, n (%)	
Malay	154 (39.7)
Chinese	151 (38.9)
Indian	79 (20.3)
Others	4 (1.1)
Marital status, n (%)	
Married	304 (78.3)
Single	79 (20.4)
Others	5 (1.3)
Education level, n (%)	
No formal education	27 (7)
Primary	93 (24)
Secondary	176 (45.4)
Tertiary, (college/university)	92 (23.6)
Working status, n (%)	
Yes	102 (26.3)
No	286 (73.7)
Dialysis Vintage (months)	78 ± 74
Post-dialysis weight (kg)	61.7 ± 14.7
Height (cm)	158 ± 9
Body Mass Index (kg/m^2)	24.6 ± 5.2

Note: Data are presented as mean ± SD unless stated as percentage (%).

Table S2: Food item listing in the prototype HD-FFQ

No.	Food Groupings	Subgroups	Total food items
**1.**	Cooked rice	-	6
**2.**	Noodles	5 subgroups:<br>(a) Rice/starch noodles<br>(b) Wheat noodles<br>(c) Instant noodles<br>(d) Pasta<br>(e) Others	10
**3.**	Cereals & products	4 subgroups:<br>(a) Biscuits<br>(b) Bread<br>(c) Bun/croissant<br>(d) Others	15
**4.**	Meat, poultry & products	-	6
**5.**	Fish, shellfish & products	-	6
**6.**	Legumes	-	4
**7.**	Vegetables	-	6
**8.**	Fruit	-	13
**9.**	Fast foods	-	4
**10.**	Milk & dairy products	-	2
**11.**	Traditional kuih	3 subgroups:<br>(a) Malay kuih<br>(b) Chinese kuih<br>(c) Indian kuih	6
**12.**	Bakery & sweets	-	4
**13.**	Snacks & finger foods	-	11
**14.**	Jam & spread	-	4
**15.**	Beverages	3 subgroups:<br>(a) Coffee/tea<br>(b) Malted drink<br>(c) Others	10
**16.**	Sauces, condiments & soups	-	9
**17.**	Health/oral nutrition supplements	-	2
		**Total food items**	**118**

Table S3: Conversion factor for the frequency of food item intake

**Frequency of intake**	**Conversion Factor**
Per day	1/1 = 1.00
Per week	1/7 = 0.14
Per month	1/30 = 0.03

Figure S1: Bland-Altman Plot for dietary intakes derived by HD-FFQ and 3DDR methods

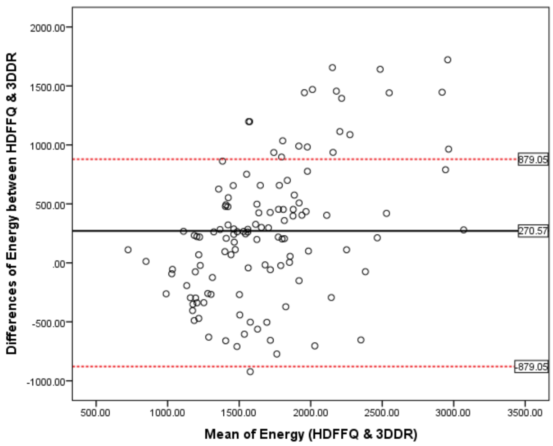

(A)

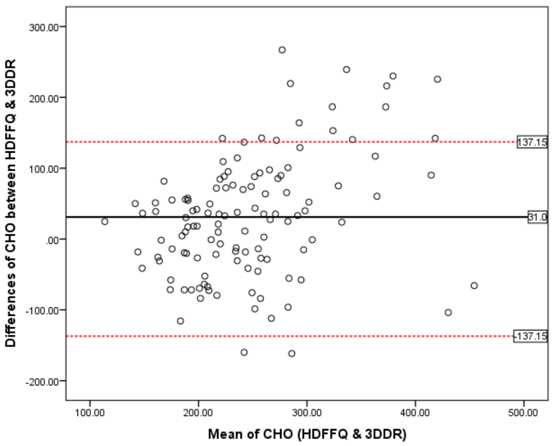

(B)

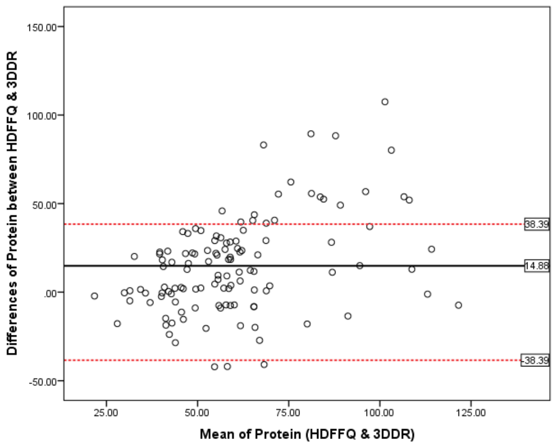

(C)

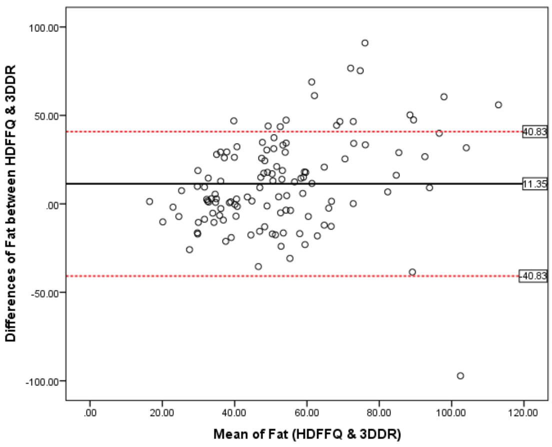

(D)

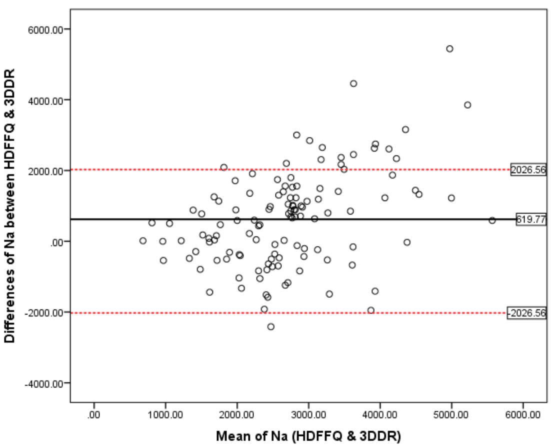

(E)

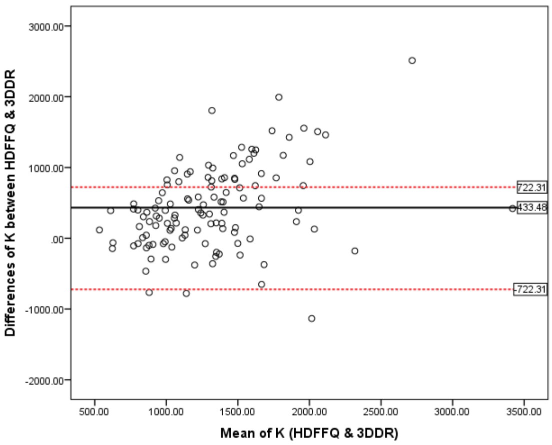

(F)

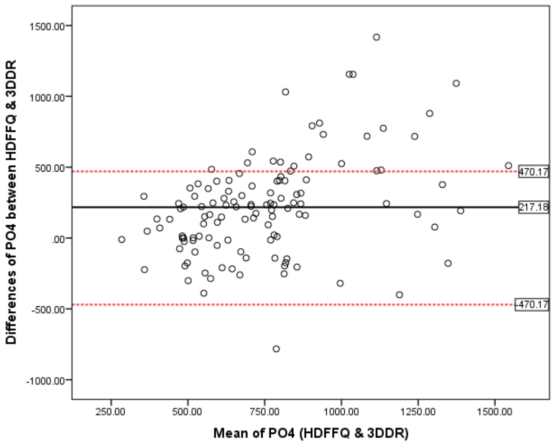

(G)

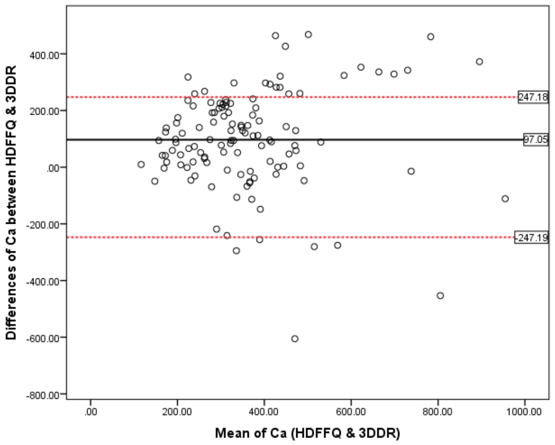

(H)

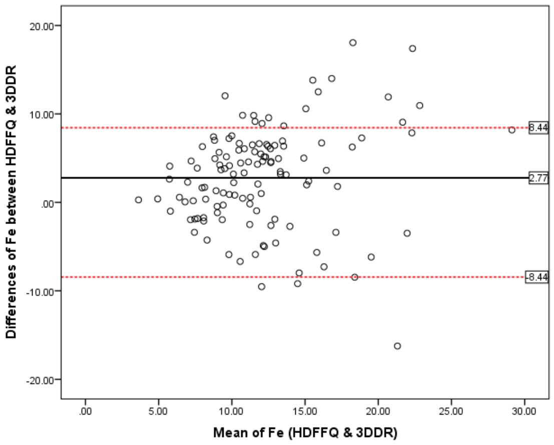

(I)

Note: Bland-Altman plots visualizing the agreement between the HD-FFQ and 3DDR to assess the intake of (A) Energy, (B) Carbohydrate, (C) Protein, (D) Fat, (E) Sodium, (F) Potassium, (G) Phosphate, (H) Calcium, and (I) Iron. For all plots, the middle line represent the mean differences between the HD-FFQ and 3DDR and limits of agreement (dotted line) indicates the 95% confidence interval (mean ± 1.96 SD). The linear regressions produced significant p-values (<0.05) for all nutrients except for calcium: p= 0.46 and iron: p= 0.05. The significant values indicates that the Bland-Altman plot is skewed (i.e. the difference in absolute intakes increased with increasing average intake, with the mean of HD-FFQ produce a higher estimates than the average 3DDR).

Table S4: Summary of comments from the expert and lay person

No.	Open comments	Commenter
**1.**	Layout easy to follow and the food items are relevant to local foods.	Nephrologist 1
**2.**	The HD-FFQ are not too long, but can consider to separate the questionnaire into two parts since patients might have short attention span.	Nephrologist 2
**3.**	Suggest to show the household measure to patients during the administration of HD-FFQ. To improve patients portion size estimation.	Dietitian 1
**4.**	May consider to translate the HD-FFQ to 'Bahasa' or 'Mandarin' language.	Dietitian 2
**5.**	Usual food item consumption is available in the HD-FFQ.	Patient 1
**6.**	Questionnaire is clear and easy to understand, but may consider to increase the font size as most of the dialysis patients are elderly.	Patient 2

Table S5: Food item listing in the final HD-FFQ

No.	Food Groupings	Subgroups	Total food items
1.	Cooked rice	-	7
2.	Noodles	5 subgroups:<br>(a) Rice/starch noodles<br>(b) Wheat noodles<br>(c) Instant noodles<br>(d) Pasta<br>(e) Others	12
3.	Cereals & products	4 subgroups:<br>(a) Biscuits<br>(b) Bread<br>(c) Bun/croissant<br>(d) Others	15
4.	Meat, poultry & products	-	7
5.	Fish, shellfish & products	-	5
6.	Legumes	-	3
7.	Vegetables	-	6
8.	Fruit	-	14
9.	Fast foods	-	5
10.	Milk & dairy products	-	2
11.	Traditional kuih	3 subgroups:<br>(a) Malay kuih<br>(b) Chinese kuih<br>(c) Indian kuih	7
12.	Bakery & sweets	-	4
13.	Snacks & finger foods	-	9
14.	Jam & spread	-	4
15.	Beverages	3 subgroups:<br>(a) Coffee/tea<br>(b) Malted drink<br>(c) Others	11
16.	Sauces, condiments & soups	-	10
17.	Health/oral nutrition supplements	-	2
		**Total food items**	**123**

Subject Code:

No.	Food	Taken (yes/no)	Frequency				Household measurements	Portion taken	Extra gravy (1 Tbsp)
			per day	per week	per month	rarely			
**Cooked Rice**									
1	White rice						1 small bowl		
2	Brown rice/Parboiled rice						1 small bowl		
3	*Nasi ayam**/ Claypot *(*Fried/roasted/steamed chicken)*						1 serving		
4	*Nasi lemak (Standard set)*						1 small bowl		
5	Fried rice						1 medium bowl		
6	*Nasi Minyak/Beriani*						1 medium bowl		
7	Porridge *(Plain/Chicken/Fish/Pork)*						1 medium bowl		
**Noodles**	***please specify the amount of extra gravy if any**								
**A**	**Rice / Starch noodles (Mee hoon/Kuey teow/Loh see fun/Tang hoon)**								
8	Fried/Dried						1 medium bowl		
9	Soup						1 medium bowl		
10	Curry						1 medium bowl		
11	*Laksa/Cantonese*						1 medium bowl		
**B**	**Wheat noodles (Yellow noodle/Wanton mee/Yee mee/Pan mee/Lor mee/Ramen/Misua/Udon)**								
12	Fried/Dried						1 medium bowl		
13	Soup						1 medium bowl		
14	Curry/*Rebus*						1 medium bowl		
15	Cantonese/Hokkien						1 medium bowl		
**C**	**Instant noodles**								
16	Fried/Dried						1 packet (m)		
17	Soup						1 packet (m)		
**D**	**Pasta (Fettucini/Spaghetti/Macaroni)**								
18	Fried/Sauce* *(*White; Carbonara / Red; Bolognaise)*						1 medium bowl		
**E**	**Others**								
19	*Chee Cheong Fun (Plain/Char siew/Prawn)*						1 piece		
**Cereals and products**									
**A**	**Biscuits**								
20	Sweet *(Cream filling/Marie/ jam, etc.)*						1 piece		
21	Salty *(Cream cracker, etc.)*						1 piece		
**B**	**Bread**								
22	White						1 slice		
23	Wholegrain/High fibre						1 slice		
**C**	**Bun/Croissant**								
24	Plain						1 piece		
25	Sweet filling/Flavoured *(Vanilla/chocolate/corn/kaya/red bean/butter/coffee, etc.)*						1 piece		
26	Savoury *(Meat filling/Chicken floss/Ba kua/French toast/Mushroom, etc.)*						1 piece		
**D**	**Others**								
27	*Roti canai (Plain)*						1 piece		
28	*Roti canai (Egg/sardine)*						1 piece		

Subject Code:

No	Food	Taken (yes/no)	Preparation Method	Frequency				Household measurements	Portion taken	Extra gravy (1 Tbsp)
				per day	per week	per month	rarely			
**D**	**Others**									
29	*Chapati*							1 piece		
30	*Thosai*							1 piece		
31	*Idli*							1 piece		
32	Oats							1 tablespoon		
33	Cereal Beverage (Eg: Nestum) *(Plain/Condensed milk)*							1 glass		
34	Cereal Beverage (Eg: Nestum) *(3 in 1)*							1 sachet		
**Meat, Poultry & Products**				***please specify amount of extra gravy if any**						
35	Chicken		Fried/stir fried/grilled/ braised/ *masak kicap*					1 matchbox		
			Curry/*rendang/lemak/sambal /satay*					1 matchbox		
			Steamed/soup					1 matchbox		
36	Beef/Mutton/Lamb		Fried/curry/ *rendang/masak lemak*/stir fried/*masak kicap/satay*					1 matchbox		
			Soup/*masak pinang*					1 matchbox		
37	Pork		Fried/stir fried/grilled/ braised/*masak kicap/*roasted					1 matchbox		
			Steamed/soup					1 matchbox		
38	Egg (with egg yolk) *(Chicken/Duck/Quail)*		Fried/omelette/ scrambled/*sambal*					1 whole		
			Curry/*gulai*					1 whole		
			Boiled					1 whole		
			Salted					1 whole		
			Steamed					1 whole		
			Preserved/ Century					1 whole		
	Egg (white only) *(Chicken/Duck/Quail)*		Fried/omelette/ scrambled/ *sambal*					1 whole		
			Curry/*gulai*					1 whole		
			Boiled					1 whole		
			Salted					1 whole		
			Steamed					1 whole		
			Preserved/Century					1 whole		
**Processed food**										
39	Nugget/Sausage/Meatball *(Chicken/Lap cheong/ Ham/Pork/Bacon)*		Fried/stir fried					1 piece		
			Steamed/Boiled/Soup					1 piece		
**Internal Organs**				***please specify amount of extra gravy if any**						
40	Beef/Chicken/Pork *(Gizzard/Liver/Intestine/ Lung/Heart)*		*Sambal/masak lemak/*fried/boiled					1 matchbox		
**Fish, Shellfish & Products**				***please specify amount of extra gravy if any**						
41	Anchovies *(Ikan Bilis)*		Fried/stir fried/*sambal*					1 tablespoon		
42	Sea fish *(Kembong/Bawal/Kerapu/ Kerisi/Merah/Parang/Salmon/Sardine/Selar/Siakap/Tenggiri, etc.)*		Fried/stir fried/*masak kicap*					1 matchbox		
			Curry/*sambal/masak lemak*					1 matchbox		
			Steamed/soup/singgang					1 matchbox		
			Canned					1 matchbox		

Subject Code:

No.	Food	Taken (yes/no)	Preparation method	Frequency				Household measurements	Portion taken	Extra gravy (1 Tbsp)
				per day	per week	per month	Rarely			
**Fish, Shellfish & Products**			***please specify amount of extra gravy if any**							
43	Freshwater fish *(Keli/Tilapia/Haruan/Patin, etc.)*		Fried/stir fried/*masak kicap*					1 matchbox		
			Curry/*masak lemak/sambal*					1 matchbox		
			Steamed/soup					1 matchbox		
44	Crab/Prawn		Fried/stir fried/*masak kicap*					1 matchbox		
			Curry/*masak lemak/sambal*					1 matchbox		
			Steamed/soup					1 matchbox		
45	Squid/Shellfish* *(Cockle/Lala/Abalone*)*		Stir fried/ *masak kicap*/braised					1 matchbox		
			Curry/*masak lemak/sambal*					1 matchbox		
			Steamed/soup					1 matchbox		
**Processed food**										
46	Fish ball/Fishcake/Squid Ball		Fried/Stir fried					1 piece		
			Boiled/steamed/soup					1 piece		
**Legumes**			***please specify amount of extra gravy if any**							
47	*Tau hoo/Tau kua/Fucuk*		Fried					1 piece		
48	*Tau hoo/Tau hoo pok/Fucuk*		Stir-fried/braised/*masak kicap*					1 piece		
49	*Tau hoo*		Soup/steamed/boiled					1 piece		
**Vegetables**			***please specify amount of extra gravy if any**							
50	Green leafy vegetable *(Spinach/Kale/Sweet potato leave/Mustard green/kangkung/Pucuk Paku/Kucai etc.)*		Stir fried/curry/*sambal*					1 tablespoon		
			Masak air/soup/boiled					1 tablespoon		
			Raw/*Ulam*					1 tablespoon		
51	Cruciferous vegetable *(Cabbage/broccoli/ cauliflower/lettuce/you mak/bak choy etc.)*		Stir fried/curry/*masak lemak*					1 tablespoon		
			Soup/boiled/tom yam					1 tablespoon		
			Raw/ *Ulam*					1 tablespoon		
52	Beans vegetable *(French/Long/Winged/ Tau geh etc.)*		Stir fried/curry/*masak lemak*					1 tablespoon		
			Raw/ *Ulam*					1 tablespoon		
53	Fruit vegetable *(Bitter gourd/Cucumber/ Pumpkin/ Baby corn/Petola/Lady finger/Brinjal/Winter melon/Plaintai/Chili, etc.)*		Stir fried/curry/*masak lemak/sambal*					1 small bowl		
			Boiled/soup					1 small bowl		
			Raw/ *Ulam*					1 small bowl		
54	Root vegetable *(Potato/Sweet potato/Yam/Carrot/Radish/ Beetroot/Sengkuang/Lotus root, etc.)*		Stir fried/braised/curry/ *Masak lemak/sambal/masak kicap*					1 small bowl		
			Fried					1 small bowl		
			Boiled/steam/soup					1 small bowl		
			Raw/ *Ulam*					1 small bowl		
55	Mixed vegies (Acar)							1 tablespoon		

Subject Code:

No	Food	Taken (yes/no)	Preparation method	Frequency				Household measurements	Portion taken	Extra gravy (1 Tbsp)
				per day	per week	per month	rarely			
**Fruit**	***please specify amount of 'Flavouring, Eg: Asam Boi/Salt' if taken together with fruit**									**Asam Boi/Salt (1 tbsp)**
56	Apple/Pear							1 piece		
57	Banana							1 medium piece		
58	Dates *(Kurma)*							1 piece		
59	Seasonal fruit *(Duku/Langsat/Rambutan)*							1 piece		
60	Durian							1seed		
61	Grapes							1 piece		
62	Guava							1 slice		
63	Mango							1slice		
64	*Nangka*							1 piece		
65	Citrus fruit *(Orange/Limau Mandarin)*							1 piece		
66	Papaya							1 slice		
67	Pineapple							1 slice		
68	Melon *(Watermelon/Honeydew)*							1 slice		
69	Starfruit							1 whole		
**Fast foods**										
70	Fried chicken *(KFC, McD, etc.)*							1 piece		
71	French fries/Potato wedges							1 small bowl		
72	Pizza							1 medium slice		
73	Burger *(Beef/Chicken/Fish)*							1 whole (m)		
74	Salad/Coleslaw							1 small bowl		
**Milk & Dairy products**										
75	Low fat milk powder *(Omega/Anlene, etc.)*							1 tablespoon		
76	Full cream milk powder *(Fernleaf/Dutchlady,etc.)*							1 tablespoon		
**Traditional *Kuih***										
**A**	**Malay *Kuih***									
77	Fried *(Pisang goreng/Cucur udang/Cucur bilis/Cucur sayur/Cekodok/Roti jala/Rempeyek/Pau Sambal/Karipap)*							1 piece		
78	Sweet *(Kuih bingka/Lempeng/Apam balik/Kuih Bakar/Kuih Ketayap/Bahulu/Kuih Kasui/Seri Muka/Kuih Koci/Kuih Talam)*							1 piece		
**B**	**Chinese *Kuih***									
79	Fried Kuih *(Eg: Yau Zha Kuih/Ham Chi Ping)*							1 piece		
80	Steamed kuih *(Pau Pork/Chocolate/Kaya/Coconut/Bak Tong Gou/Ang Koo Kueh)*							1 piece		
81	Steamed Dumpling *(Rice dumpling, Bak Zang/Loh mai kai/Loh mai fan)*							1 piece		
**C**	**Indian *Kuih***									
82	Fried *(Vadai/Samosa)*							1 piece		
83	Steamed *(Idiyappam/Puttu)*							1 piece		
**Bakery & Sweets**										
84	Cakes *(Plain/Sponge cake)*							1 slice		
85	Cakes, Cream/Flavoured *(Banana/Carrot/Chocolate/Red velvet/Tiramisu/Butter/Cheese, etc.)*							1 slice		
86	Doughnut *(Plain/Sugar/Icing)*							1 piece		
87	Jelly/Agar/Dadih							1 piece		

Subject Code:

No	Food	Taken (yes/no)	Preparation method	Frequency per day	per week	per month	rarely	Household measurements	Portion taken	Extra gravy (1 Tbsp)
**Snacks & Finger Foods**										
88	*Muruku*							1 piece		
89	*Keropok (Ikan/Prawn/Lekor)*							1 piece		
90	*Popiah*		Fried					1 piece		
			Basah					1 piece		
91	*Dim sum*		Fried					1 piece		
			Steamed					1 piece		
92	*Sushi*							1 piece		
93	Sandwich *(Egg/Chicken/Ham/Tuna)*							1 set (2 slices of bread)		
94	Yong tau foo *(Lady finger/Bitter gourd/Brinjal/Chilli)*		Fried					1 piece		
			Boiled					1 piece		
95	Yong tau foo *(Tau hoo/Tau pok)*		Fried					1 piece		
			Boiled					1 piece		
96	*Kebab (Beef/Chicken/Fish/Mutton)*							1 piece		
**Jam & Spread**										
97	Butter							1 teaspoon		
98	Fruit jam *(Pineapple/strawberry, etc.)*							1 teaspoon		
99	Kaya							1 teaspoon		
100	Margarine		Salted					1 teaspoon		
			Unsalted					1 teaspoon		
	**Beverages**									
**A**	**Coffee/Tea**									
101	'Tarik'/ 'C'/Canned/ milk							1 glass		
102	O (with sugar)							1 glass		
103	3 in 1							1 sachet		
104	2 in 1 (with sugar only)							1 sachet		
105	With Non-dairy creamer							1 glass		
**B**	**Malted Drink**									
106	Condensed milk/Sugar/Plain							1 glass		
107	3 in 1							1 sachet		
**C**	**Other drinks**									
108	Cordial/Syrup							1 glass		
109	Fresh juice/Barley/Herbal drink							1 glass		
110	Sugar Sweetened Beverage *(Soy bean milk, etc.)*							1 glass		
111	Carbonated/Isotonic drink *(100 plus/coke, etc.)*							1 can		
**Sauces, Condiments & Soups**										
112	*Budu/Cincaluk*							1 tablespoon		
113	Bottled Sauce *(Chilli/Tomato)*							1 tablespoon		
114	Coconut *Chutney*							1 tablespoon		
115	Tomato/Mint *Chutney*							1 tablespoon		
116	*Sambal tumis*							1 tablespoon		
117	*Sambal belacan*							1 tablespoon		
118	Soy sauce (Light/dark)							1 tablespoon		
119	Chilli *(Pickled/Cutted/with soy sauce)*							1 tablespoon		

Subject Code:

No.	Food	Taken (yes/no)	Preparation method	Frequency				Household measurements	Portion taken	Extra gravy (1 Tbsp)
				per day	per week	per month	rarely			
**Sauces, Condiments & Soups**										
120	Dhal/ Green gram		Curry (Santan)					1 tablespoon		
			Curry (No santan)					1 tablespoon		
	**Soup (only)**									
121	Clear soup *(Herbal/Vegetable soup/Chicken/Fish/Fishball/Meatball, etc.)*							1 small bowl		
**Health / Oral Nutrition Supplements**										
122	Oral Nutrition Supplements; Ready To Drink *(Please specify:)*							1 bottle/packet		
123	Oral Nutrition Supplements; Powder *(Please specify:)*							1 scoop/sachet		
	Others:									

Table S6: Snapshot of the HD-FFQ Form

Table S7: Identification of misreporters according to EI: BMR Category

EI:BMR Category	3DDR (n=152)	HD-FFQ (n=152)
**Under-reporter**	16 (11%)	11 (7%)
**Acceptable-reporter**	136 (89%)	133 (88%)
**Over-reporter**	-	8 (5%)

Note: Data are presented as number and percentage; n (%).

Abbreviations: EI: BMR: Energy Intake: Basal Metabolic Rate, 3DDR: 3-Day Diet Recalls, HD-FFQ: Hemodialysis Food Frequency Questionnaire
